# Efficacy and Complications of the Re-Adjustable Male Sling System for Stress Urinary Incontinence after Radical Prostatectomy

**DOI:** 10.3390/jcm11226764

**Published:** 2022-11-15

**Authors:** Liang-Wei Chiu, Wen-Chi Chen, Po-Fan Hsieh, Yung-Hsiang Chen, Chi-Ping Huang

**Affiliations:** 1 Department of Urology, China Medical University Hospital, Taichung 40447, Taiwan; 2 Graduate Institute of Integrated Medicine, College of Chinese Medicine, China Medical University, Taichung 40402, Taiwan; 3 School of Medicine, College of Medicine, China Medical University, Taichung 40402, Taiwan; 4 Department of Psychology, College of Medical and Health Science, Asia University, Taichung 41354, Taiwan

**Keywords:** male sling, prostate cancer, radical prostatectomy, stress urinary incontinence

## Abstract

The aim of this study was to investigate the outcomes of re-adjustable male slings in pa-tients with postoperative stress urinary incontinence (SUI) following radical prostatectomy (RP). We retrospectively analyzed 18 patients with SUI following RP for prostate cancer, who were treated with re-adjustable male slings from January 2016 to December 2021. The clinical outcomes were evaluated based on daily pad usage and urodynamic studies, both preoperatively and post-operatively. The degree of SUI was categorized as either mild, moderate, or severe. Success was defined as no more pad use or significantly decreased pad use. Complications were classified ac-cording to the Clavien–Dindo system. The mean age of patients was 70.4 ± 5.9 years, and 61.1% of cases were diagnosed as locally advanced prostate cancer. Mild, moderate, and severe SUI were reported as 33.3%, 50.0%, and 16.7%, respectively. The average daily pad use after RP was 3.3 pads and there was a significant reduction in the number of daily pads used after the re-adjustable male sling procedure (3.3 vs. 1.3; *p* = 0.002). Overall, the operation was considered successful in 11 pa-tients (61.1%), 1 case showed improvement (5.6%), and it was considered unsuccessful in 6 patients (33.3%). Impressively, in the severe incontinence subgroup (three patients), one (33.3%) had im-provement and one (33.3%) had success. Transient perineal discomfort after the operation was the most common complaint. However, two patients had severe complications with bowel injury during sling implantation (Clavien–Dindo grade III). The re-adjustable male sling system was an efficient surgical treatment option for post-RP SUI, even in a locally advanced disease dominant population. One should pay attention to the complication of bowel perforation during surgery.

## 1. Introduction

Postoperative stress urinary incontinence (SUI) has a negative effect on the quality of life for prostate cancer patients undergoing radical prostatectomy (RP) [1,2], in which external urethral sphincter function may be compromised. The rate of SUI after RP ranges from 1 to 40% [3,4]. Compared with open retropubic radical prostatectomy (RRP), robotic-assisted laparoscopic radical prostatectomy (RALRP) has been proven to decrease the incontinence rate after surgery [5]. However, the overall incidence of SUI still ranged from 8 to 11% at 1 year after RALRP [5]. The severity of SUI can be influenced by patient characteristics, surgeon experience, the surgical method used, the definition of incontinence, the length of follow-up, and the tools used for data collection [5]. Nevertheless, management of post-op SUI for both surgeries remains an important issue. The initial treatment for postoperative SUI includes pelvic floor muscle training and medical therapy such as Duloxetine, a combination of norepinephrine and serotonin reuptake inhibitors. Conservative treatments, such as Kegel exercises and pelvic floor muscle training have been shown to improve recovery of continence in patients undergoing RP [6,7,8,9]. Duloxetine was reported to have an adverse event rate of 15.2%, and included side effects such as fatigue and nausea [10]. The long-term results for pharmacological treatment also seem disappointing [10]. There was a lack of randomized control-trails regarding medical treatment for SUI and surgical treatment is becoming urgent. The gold standard for surgical treatment is an artificial urinary sphincter, especially for severe SUI [11]. Other surgical options, including urethral bulking agents, fixed male slings, and adjustable male slings, have variable efficacy in patients with mild to moderate SUI [11,12]. In this study, we aimed to report our experience of SUI treatment with a re-adjustable male sling in patients who underwent RP.

## 2. Materials and Methods

In this study, 18 patients from a single medical center in Taichung, Taiwan with SUI at least one year after RP or RALRP between January 2016 and December 2021 were retrospectively analyzed. They were treated with re-adjustable male slings (NEOMEDIC Contasure Remeex Male Adjustable System). All patients were followed for at least 12 months after their operation. The severity of their SUI was defined as either mild (≤2 pads/day), moderate (3–5 pads/day), or severe (>5 pads/day) [13]. Three months after the operation for re-adjustable male sling implantation, we evaluated the patient’s daily pad usage and conducted a urodynamic study; this was also performed preoperatively. The time taken for male sling adjustment and any complications were also recorded. The clinical efficacy was evaluated by a reduction in the number of daily pads used after male sling implantation. The operation was considered a success if the patient wore no pads or used security pads to prevent a bit of unpredictable urine leakage in rare situation but remained dry most days. The condition was considered improved if the number of daily pads used decreased by >50%, while the operation was considered unsuccessful if the number of daily pads used decreased by <50%. Complications were classified using the Clavien–Dindo system [14]. 

All statistical analyses were performed using IBM SPSS Statistics for Windows [15,16], Version 25.0 (IBM Corp. Released 2017. Armonk, NY, USA: IBM Corp.). The research ethics committee of China Medical University and Hospital approved this study (approval no. CMUH111-REC3-063).

## 3. Results

Table 1 depicts the baseline characteristics of 18 patients with prostate cancer and SUI after RP who then underwent a sling operation. The mean age of the patients was 70.4 years, and 61.1% of cases (11 patients) were diagnosed with locally advanced prostate cancer. There were 17 RALRPs and 1 open retropubic prostatectomy. All RALRPs were performed using an anterior approach without Retzius sparing. Mild, moderate, and severe SUI were reported as 33.3%, 50.0%, and 16.7%, respectively. The average daily pad use after RP was 3.3 pads before the first sling procedure. All patients received medical therapy such as Imipramine before the male sling procedure, which revealed no significant improvement. The average time after the RP until the sling operation was performed was 23.4 ± 12.39 months (ranged from 12 to 50 months).

Table 2 shows a significant reduction in the number of daily pads used after the sling operation (3.3 vs. 1.3; *p* = 0.002). Preoperative and postoperative maximal cystometric capacity, maximum flow rates, and postvoid residual volumes were similar (*p* = 0.433, *p* = 0.052, and *p* = 0.594, respectively).

Re-adjustable male sling implantation was reported as successful in 11 patients (61.1%), 1 patient had improvement (5.6%), and 6 patients (33.3%) were considered unsuccessful. Notably, in the severe incontinence subgroup (three patients), one (33.3%) had improvement and one (33.3%) had success (Table 3). No patients needed more pads after re-adjustable male sling implantation was performed.

Three patients (16.7%) had an episode of urinary retention. Three patients (16.7%) had an episode of mesh infection. Two patients (11.1%) were complicated with bowel injuries during sling implantation (Table 4).

One patient had device failure (43 months after the first implantation) and underwent reimplantation of a new re-adjustable male sling with a successful outcome.

## 4. Discussion

Our results show that the re-adjustable male sling system was an efficient surgical treatment option for post-RP SUI. With a median follow-up time of 11.5 months, 66.7% of our cases had an improved or successful outcome. A similar outcome was observed in the severe SUI subgroup. Even if device failure developed, a satisfactory outcome may still be observed after reimplantation of a new re-adjustable sling. Transient perineal discomfort was the most common complaint. However, severe complications (Clavien–Dindo classification grade III or greater) should be treated quickly, such as bowel injury.

SUI following RP impairs quality of life after the operation [17]. It is related to sphincteric insufficiency [5], and it can gradually improve 6 to 12 months after the RP operation [18]. For moderate to severe SUI, an artificial sphincter is considered the gold standard treatment. However, it is associated with significant complications, including infection, erosion, mechanical failure, and urethral atrophy [19]. The re-operation rate varies from 14.8% to 44.8% [19]. Furthermore, some patients worry about device failure and their ability to manipulate the switch. Due to the above, less invasive alternatives, such as the Remeex Male Adjustable System, have been developed. Furthermore, Del Giudice et al. reported that male slings were the most common incontinence procedure and the first-choice treatment for up to 50% of patients in the United States [20].

In a recent study, the Remeex Male Adjustable System reported an 89.36% improvement and/or cure rate for mild to moderate SUI [21]. In another systemic review, the continence rate was up to 84.3% following use of the Remeex Male Adjustable System [22]. Although the male sling has been indicated for mild to moderate SUI, Marquez-Sanchez et al. observed that the direct negative association between grade of SUI and effectiveness was not significant. The Remeex Male Adjustable System has been reported to be effective, regardless of SUI severity [21]. Navalón-Monllor et al. reported that all 24 patients with post-prostatectomy severe SUI that received a Remeex sub-urethral tension-adjustable sling remained dry after the operation, and only nine of them (39%) occasionally needed a safety pad if they did intense exercise [23]. In our study, with a median follow-up of 11.5 months, 66.7% of cases were considered successful or improved, while 33.3% of cases remained unchanged. The continence rate was lower than that observed in previous studies, and this might be related to the shorter follow-up. As the Remeex Male Adjustable System is re-adjustable, the effect of the sling can be improved by re-adjusting. We also observed that it provided a similar outcome in the severe SUI subgroup compared with the other groups. Previous literature reported that 34% to 91% of cases required at least one readjustment [21,23]. Our study showed a similar re-adjustment rate (55.6%).

The complication rate for the Remeex Male Adjustable System was about 35.8% based on a previous study [24]. Most patients complained of perineal discomfort or pain, which was easily treated with oral medications. Infection, perineal hematoma, and intraoperative bladder perforation were other possible complications [22]. Uneventful bladder perforation during surgery was not rare and might be up to 10.64% [21]. In our study, transient perineal discomfort was the most common complaint, which was easily treated with oral medications. Three patients had an episode of urinary retention after the operation, for which urethral sounding or temporary urethral catheter indwelling was needed. Three patients had an episode of mesh infection, and they were all successfully treated with a complete course of antibiotic treatment without the need to remove the mesh. Two patients had severe complications with bowel injuries occurring during sling implantation. The slings were immediately removed after signs of bowel injury were detected, which was within 2 days in both cases. One patient recovered smoothly after empiric antibiotic and conservative treatment. However, the other patient had two sigmoid colon perforations and one ileum perforation 60 cm from the ileocecal valve, and underwent an operation to repair the perforation (Table 4). In addition, two cases had bowel perforation which has been rarely reported in other studies. All cases enrolled in the current study underwent transperitoneal approach RP. The peritoneum was incised while developing the space of Retzius, and it was not routinely repaired at the end of the surgery. The bowel therefore had the chance to migrate to the retropubic space, which might increase the risk of bowel perforation when a wire-passing needle was retropubically inserted, rubbing the inner surface of the pubic bone. Peritoneum repair at the end of RP for higher risk post-operative SUI patients or image surveillance, such as magnetic resonance imaging (MRI), before sling surgery could be considered to decrease the risk of bowel perforation.

Most published studies on the use of the Remeex Male Adjustable System in patients with SUI following RP were based in European medical centers [13,21,22,23,24,25,26]. To the best of our knowledge, this report is the first study on the efficacy of the Remeex Male Adjustable System in an Asian population. About 61.1% of our cases were diagnosed as locally advanced prostate cancer at pT4 or pN1 stages. It is important to avoid a positive surgical margin, however it can be difficult in RP for locally advanced prostate cancer to do so whilst also preserving the neurovascular bundles (NVB), external urethral sphincter, or bladder neck, leading to a higher rate of SUI [27,28,29]. Our results showed that in a locally advanced disease dominant population, the re-adjustable male sling system was an efficient surgical treatment option for post-RP SUI. Even if device failure develops, it can still provide a satisfactory outcome after reimplantation.

Limitations of this study included the use of a single-institution, and single surgeon experience, as well as the small number of patients, and relatively short follow-up time. There was also a lack of quality-of-life assessment and pad weight testing. External validation of the Remeex Male Adjustable System in a prospective, large-scale study is needed.

## 5. Conclusions

The re-adjustable male sling system (Remeex Male Adjustable System) is an efficient surgical treatment option for post-RP SUI, regardless of SUI severity. It may still provide a satisfactory outcome after re-implantation if there is device failure. Transient perineal discomfort after the operation was the most common complaint, which was easily treated with oral medications. Severe complications, such as bowel injury and device infection, were rare but should be considered and treated quickly.

## Figures and Tables

**Table 1:** Baseline patient characteristics of cases treated with male sling (n = 18).

Race, n (%)	
Asian	18 (100)
Age, yr, mean ± SD (median; range)	70.4 ± 5.9 (69.5; 60–79)
BMI, kg/m^2^, mean ± SD (median; range)	26.2 ± 4.3 (26.2; 19.6–32.8)
iPSA, ng/mL, mean ± SD (median; range)	23.8 ± 28.5 (12.7; 4.2–99.0)
Gleason score of prostate biopsy, n (%)	
6	1 (5.6)
7	11 (61.1)
8	1 (5.6)
9	5 (27.8)
TNM stage of image, n (%)	
iTx	1 (5.6)
iT2	4 (22.2)
iT3a	7 (38.9)
iT3b	5 (27.8)
iT4	1 (5.6)
iNx	1 (5.6)
iN0	14 (77.8)
iN1	3 (16.7)
TNM stage of pathology, n (%)	
pT2	7 (38.9)
pT3a	4 (22.2)
pT3b	5 (27.8)
pT4	2 (11.1)
pN0	13 (72.2)
pN1	5 (27.8)
Surgical method, n (%)	
Robotic-assisted laparoscopic radical prostatectomy	17 (94.4)
Open retropubic radical prostatectomy	1 (5.6)
Nerve sparing, n (%)	
None	12 (66.7)
Unilateral	3 (16.7)
Bilateral	3 (16.7)
Previous TURP, n (%)	4 (22.2)
Previous pelvis radiation, n (%)	4 (22.2)
Previous medical treatment, n (%)	18 (100)
Time to sling, months, mean ± SD (median; range)	23.4 ± 12.4 (17.5; 12–50)
Post-RP daily pad use after at least 12 months follow-up, mean ± SD (median; range)	3.3 ± 1.8 (3; 1–8)
SUI severity, n (%)	
Mild (≦2 pads/day)	6 (33.3)
Moderate (3–5 pads/day)	9 (50.0)
Severe (>5 pads/day)	3 (16.7)
Second or more regulation, n (%)	10 (55.6)
Time to second regulation, months, mean ± SD (median; range)	4.0 ± 3.6 (3; 1–13)
Times of adjustment, mean ± SD (median; range)	1.2 ± 1.6 (1; 0–6)
Follow-up time after male sling surgery, months, mean ± SD (median; range)	18.4 ± 15.4 (14.0; 3–70)

yr: years; SD: standard deviation; BMI: body mass index; iPSA: initial prostate-specific antigen; TNM: tumor-node-metastasis; TURP: transurethral resection of prostate; RP: radical prostatectomy; SUI: stress urinary incontinence.

**Table 2:** Preoperative and last follow-up postoperative daily pad use and voiding parameters.

	Preoperative	Last Follow-Up Postoperative	*p* Value
Pad use per day	3.3 ± 1.8 (3; 1–8)	1.3 ± 1.5 (1.0; 0–5)	0.002
Urodynamic parameter			
MCC, mL	161.6 ± 109.5 (172.9; 22.0–330.2)	167.1 ± 106.2 (151.7; 10.3–340.9)	0.433
Qmax, mL/min	19.6 ± 12.4 (16.4; 2.6–55.0)	15.4 ± 9.2 (14.1; 4.6–40.9)	0.052
PVR, mL	9.3 ± 16.5 (0; 0–60.0)	13.3 ± 25.0 (0; 0–90.0)	0.594

MCC: maximum cystometric capacity; Qmax: maximum flow rate; PVR: postvoid residual urine volume.

**Table 3:** Subgroup analysis.

	Success	Improved	Unsuccess
Mild incontinence (n = 6), n (%)	3 (50)	0 (0)	3 (50)
Moderate incontinence (n = 9), n (%)	7 (78)	0 (0)	2 (22)
Severe incontinence (n = 3), n (%)	1 (33)	1 (33)	1 (33)

**Table 4:** Postoperative complications (n = 18).

The Clavien–Dindo Classification		n (%)
Grade II	Mesh infection	3 (16.7)
Grade IIIa	Urine retention	3 (16.7)
Grade IIIb	Bowel injury during sling implantation	2 (11.1)

## Data Availability

All data are available upon request to the corresponding author.
